# Communication, everyday life and technology from the perspectives of children with life-limiting or life-threatening conditions: a qualitative study

**DOI:** 10.1136/bmjpo-2026-004998

**Published:** 2026-09-15

**Authors:** Linda Johanne Martinsen, Heidi Holmen, Simen Alexander Steindal, Anette Winger

**Affiliations:** 1 Department of Nursing and Health Promotion, Faculty of Health Sciences, Oslo Metropolitan University, Oslo, Norway; 2 Oslo University Hospital, Oslo, Norway; 3 VID Specialized University, Oslo, Norway; 4 Lovisenberg Diaconal Hospital, Oslo, Norway

**Keywords:** Child, Palliative Care, Qualitative research, Technology

## Abstract

**Background:**

Children with life-limiting or life-threatening conditions who are eligible for paediatric palliative care often have complex care needs. However, they prefer to live as normally as possible, which necessitates coordinated and multidisciplinary support that allows them to be at home rather than in a hospital. Advancements in technology have the potential to support home-based care, yet little is known about children’s own communication needs, their views on home-based care and the possibilities of technology to support communication. Previous research has primarily focused on the views of parents and healthcare professionals. Consequently, this study aimed to explore what children with life-limiting or life-threatening conditions find important in communication with healthcare professionals and others and the role of technology in daily life.

**Methods:**

This qualitative study employed an exploratory design and used individual semi-structured interviews with children eligible for paediatric palliative care. Seven children aged 8–15 years with life-threatening or life-limiting conditions participated. The data were analysed using reflexive thematic analysis.

**Results:**

Three themes were developed: (1) understanding technology through daily life rather than in the frame of healthcare, (2) technology has the potential to be a digital bridge to normalcy and social belonging and (3) seeking information and safety through in-person meetings.

**Conclusion:**

Children with life-limiting or life-threatening conditions place a high value on in-person communication with healthcare professionals, emphasising the need for safety, predictability and clear information, particularly when medical procedures are involved. The findings highlight gaps in children’s involvement in decision-making and underscore the importance of including children’s perspectives when developing communication strategies and technologies to support home-based paediatric palliative care. These children primarily associated technology with gaming and everyday social interactions. They had little experience with technology in their healthcare follow-up and were unfamiliar with possibilities for use in the context of communication with healthcare professionals.

**WHAT IS ALREADY KNOWN ON THIS TOPIC**

- Children’s communication needs and perspectives on technology in home-based paediatric palliative care remain underexplored, as existing knowledge primarily reflects the views of parents and healthcare professionals.

**WHAT THIS STUDY ADDS**

- Children with life-limiting or life-threatening conditions associated technology mainly with everyday social interaction rather than healthcare and reported limited involvement in decisions about their care. They emphasised the importance of maintaining social connections and receiving clear, in-person information from healthcare professionals, particularly before medical procedures.

**HOW THIS STUDY MIGHT AFFECT RESEARCH, PRACTICE OR POLICY**

- Children’s perspectives can provide valuable guidance for developing communication approaches and technology-supported care, and they highlight the importance of providing children with understandable information and opportunities to express their views.

## Introduction

 The number of children living with life-limiting or life-threatening (LL/LT) conditions eligible for paediatric palliative care (PPC) is increasing and is estimated to affect approximately 21 million children worldwide.^1 2^ Due to advances in medical treatment, many of these children are living with their conditions for long periods of time with uncertain perspectives due to deterioration of their conditions, resulting in prolonged and demanding care situations and requiring continuous support and comprehensive care.^3^ Comprehensive PPC should include a broad multidisciplinary team of healthcare professionals across care settings, addressing the needs of children throughout the various phases of PPC.^4^ Effective communication between families and healthcare professionals is fundamental to this collaboration.^4^ However, families experience unmet needs, such as service delivery and care coordination, care and bereavement support, practical and daily living support and communication and information,^5^ which might lead to the responsibility of parents to coordinate information and care in PPC settings.^6^ Clear and consistent communication supports shared understanding, facilitates continuity across services and ensures that the child’s and the family’s perspectives inform clinical decision-making.^7–9^

Many children with LL/LT conditions often have a strong desire to spend as much time as possible at home, in familiar and safe surroundings.^10 11^ For these children, the home environment often represents normalcy, belonging and the opportunity to maintain everyday routines, such as attending school, spending time with family and engaging in leisure activities.^11–13^ Staying at home and minimising hospital admissions and outpatient visits align with the core principles of PPC, which emphasise quality of life, autonomy and participation.^4^ Receiving home-based care can ease the burden on the child and the family while enhancing their sense of control and ability to cope with an otherwise unpredictable daily life.^11 14^

Advancements in technology have opened new possibilities for supporting home-based care, facilitating remote monitoring of health status, communication with healthcare professionals and guidance without the need to attend in person.^15 16^ Health technology, in this context, includes a wide understanding, from home-based medical equipment and sensors to video consultations and communications systems.^17^ Previous research has indicated that families and healthcare professionals perceive health technologies as having substantial potential to support home-based care, particularly by enhancing the accessibility, continuity and flexibility of follow-up, supporting families’ preferences to remain at home.^15 16 18^ However, the literature has largely focused on the perspectives of parents^19 20^ and healthcare professionals,^21^ who often describe these technologies as facilitating coordination, monitoring and reduced hospital visits. The emphasis on adult perspectives has led to a knowledge gap regarding children’s views on the use of technology in communication with healthcare professionals, leaving their experiences and needs under-represented in the PPC literature.^22 23^ Including children’s voices is in line with the principles of the United Nations Convention on the Rights of the Child^24^ and is essential for developing comprehensive care that truly reflects children’s needs and preferences.^25 26^ Children may offer unique insight into their needs living with an LL/LT condition and how they experience the use of technology in their follow-up. They could contribute thoughts about what good care is for them, what a meaningful life is for them and how they prefer to communicate with healthcare professionals. Listening to children’s voices can provide a nuanced and comprehensive understanding of what is needed to support high-quality care in general and home-based care.

Asking the children about their views, this study seeks to contribute new knowledge that can inform the development of tailored and child-friendly home-based care models. We aimed to explore what children with LL/LT conditions find important in communication with healthcare professionals and others, and the role of technology in daily life.

## Method

### Study design

This study employed a qualitative exploratory design using individual semi-structured interviews with children with LL/LT conditions.

### Study setting

The research was conducted within the broader project *Health Technology in Home-based Paediatric Palliative Care* (Children in Palliative Care (CHIP) homeTec), which explores the possibilities of health technology aimed at supporting communication among children, families and healthcare professionals involved in home-based PPC.^6 19^ The framework for the CHIP homeTec project is based on family-centred theory, which emphasises collaboration with families throughout the care process, recognises their expertise and promotes individualised support that addresses the holistic needs of both the child and the family.^7^

### Recruitment and participants

Recruitment of children was pursued through an open online survey targeting the parents in PPC conducted as part of the CHIP homeTec project, in which the parents were asked if their children with an LL/LT conditions would be interested in participating in an individual interview. Additionally, an information leaflet regarding the study was provided through paediatric departments in hospitals in different health regions in Norway, user organisations and departments in primary healthcare. The information leaflet had a quick response code through which parents and children could register their interest in participation. With consent from parents and children, healthcare professionals could register interest in participation on behalf of the children. When a child was registered, the first author (LJM) contacted the parents via email and/or phone, providing written and oral information about the study. If the parents found their child eligible, they discussed and clarified their participation with their child.

Convenience sampling was used, based on specific inclusion criteria specifying children aged 8–18 years with an LL/LT condition to participate. Participants had to be capable of taking part in an interview conducted in Norwegian and of being able to express themselves verbally or through alternative or supplementary communication (eg, communication boards or pictorial symbols).

For children under 12 years old who agreed to participate, the parents provided digital consent on their behalf. For children between 12 and 16 years old, both the child and the parents signed a consent form. Before the individual interviews, digital written informed consent was obtained from the children and from one of their parents. The children who participated received age-appropriate oral and written information about the study. In accordance with the conditions set by the ethics committees, we were not permitted to collect data regarding the duration of the child’s condition, receipt of palliative care or similar clinical details.

Seven children, aged 8–16 years, with various LL/LT conditions, all residing in Western Norway were included (see table 1). The children received different types of services in primary and specialised healthcare. Some also received support from PPC teams that tailored care based on their individual needs. All children were in a stable phase of their illness trajectory, and none were considered close to the end-of-life stage at the time of study participation.

**Table 1:** The age distribution and life-limiting or life-threatening condition categories of the participating children (n=7)

Participant characteristics	n=7
Age 8–11	3
Age 12–16	4
Category 1: Life-threatening conditions for which curative treatment may be feasible but can fail	2
Category 2: Conditions where premature death is inevitable	–
Category 3: Progressive conditions without curative treatment options	3
Category 4: Irreversible but non-progressive conditions causing severe disability leading to susceptibility to health complications and likelihood of premature death	2

### Data collection

Data were generated through individual interviews with children with LL/LT conditions. First, an interview guide with open-ended and probing questions, supported by pictures, was developed to facilitate dialogue during the interviews. The interview guide was distributed to all members of the CHIP research network, giving them the possibility for feedback on the relevance, clarity and comprehensibility of the questions. The CHIP network comprises parents of children with LL/LT conditions, representatives from user organisations, healthcare professionals and researchers with clinical and research experience in PPC. The researchers possess knowledge of diverse research methods, including qualitative research. The interview guide was piloted by LJM and subsequently revised with adjustments to wording and concepts to better align with the children’s language and understanding of the topics. The revisions specifically concerned the term health technology, which was adapted to technology and/or digital technology, and home-based care, referred to as follow-up (online supplemental file 1). As the revisions of the interview guide were based on wording, aiming to improve clarity and age-appropriateness, the pilot interview was considered appropriate for inclusion in the study.

All interviews were conducted between January 2024 and June 2025 and lasted 20–43 (median 25) min. LJM is a PhD candidate, a paediatric occupational therapist by training, and experienced with psychoeducational and supportive conversations with children with LL/LT conditions. She conducted all the interviews, and her experience was used to facilitate a safe environment and atmosphere during the interviews. The interviews were conducted at the location preferred by the children and their parents. The children were offered the option of bringing a trusted adult or friend to the interviews. The pilot interview was conducted in the child’s home, with one of the child’s parents present in the same room. The remaining six interviews were conducted at the children’s schools. One of the children brought a friend, who was also a child, while the other children came alone to the interviews. Parents were not given specific guidance or instructions regarding what information the children received about the study in advance or how the interviews should be presented to them. At the start of every interview, the children received age-appropriate information from LJM about the study, its purpose and an introduction to the interview topics. The children were encouraged to speak freely, and it was emphasised that no answers were wrong. All interviews were audio-recorded. The children and the parents were encouraged to contact LJM following the interview if any questions, reactions or concerns should arise afterwards.

### Data analysis

The transcribed interview data were analysed using reflexive thematic analysis.^27^ This approach was chosen for its flexibility and was regarded as suitable for exploring the children’s perspectives. The analysis was conducted inductively, with a semantic focus on exploring the meaning on an explicit level. Table 2 describes the six phases of the analysis based on Braun and Clarke’s framework of reflective thematic analysis.

**Table 2:** Illustration of the analysis process

Braun and Clarke’s six phases	Description of how the analysis was conducted
1. Familiarisation with the data	All interview data were manually transcribed by the first author, who subsequently read the transcripts multiple times to achieve deep immersion. Initial impressions, analytical reflections and spontaneous thoughts were noted directly on printed transcripts to capture early interpretive insights into the children’s descriptions.
2. Generating initial codes	Systematic coding was conducted using NVivo (QSR International, Victoria, Australia). Codes were developed from meaningful segments at the sentence and paragraph levels. As coding advanced, shared patterns across the dataset were identified, capturing both commonalities and notable differences in the children’s views.
3. Searching for themes	Candidate themes were constructed through iterative engagement with the coded data, involving grouping and regrouping of codes into broader patterns of meaning. Reflexivity was emphasised by continuously questioning how researchers’ assumptions and positionality shaped interpretive choices.
4. Reviewing themes	Candidate themes underwent several rounds of refinement. During this process, some themes were reorganised, split into subthemes or merged to more accurately reflect the complexity and nuance of the dataset. Regular memo-writing and discussions among the research team supported critical reflection and coherence. We actively sought to incorporate diverse perspectives and critically reflected on how our preconceptions shaped the research process. We did not seek consensus, and differences in interpretation among us were explored through discussion and reflexive dialogue that enabled us to move beyond our preconceptions. Rather than seeking data saturation, consistent with reflexive thematic analysis, we focused on developing themes that provided a rich and meaningful account of the children’s perspectives and adequately addressed the research aim.
5. Defining and naming themes	Final themes were clearly defined, capturing the essence of the children’s views in relation to the study’s aim. The refinement focused on ensuring internal coherence within themes and meaningful distinctions between them.
6. Producing the report	Illustrative quotes were selected to exemplify each theme, enriching the thematic narrative and ensuring that the children’s voices remained central in the presentation of findings. The final analytical narrative was grounded in reflexive engagement and the systematic exploration of patterns across the dataset.

### Preunderstanding

All authors have backgrounds as healthcare professionals. LJM has 15 years of experience in specialised habilitation care for children with and without the need for PPC. Her experience includes working with children with LL/LT conditions, for example, clinical communication and examination of the children, and through guidance and group services for guardians and siblings. This extensive experience has provided insight into the emotional and practical challenges that children and families face. Her experience has formed an in-depth understanding of the importance of family involvement in care. SAS, HH and AW are all registered nurses who have clinical and/or significant experience in qualitative research in children, palliative care, critically ill patients and health technology. All authors are members of the Norwegian national research network CHIP and are involved in the CHIP homeTec research project.

### Patient and public involvement

Parents of children with LL/LT conditions and representatives from relevant patient organisations were involved in developing the interview guide to ensure that the questions were meaningful, appropriate and aligned with family priorities. Healthcare professionals and user organisations also contributed to participant recruitment by disseminating study information to eligible families. No patients or members of the public were involved in the data collection, data analysis or preparation of the manuscript.

### Ethical considerations

The study was conducted in accordance with the Declaration of Helsinki and relevant national regulations. Confidential handling of all personal data was ensured through encrypted data collection and safe storage in a service for sensitive data.

All children and their parents received oral and written information about the study’s purpose, including the potential benefits and possible consequences of participation. They were informed that participation was voluntary and that they could withdraw at any time without providing a reason.

## Results

Three themes were developed: (1) understanding technology through daily life rather than in the frame of healthcare, (2) technology has the potential to be a digital bridge to normalcy and social belonging and (3) seeking information and safety through in-person meetings.

### Understanding technology through daily life rather than in the frame of healthcare

When the children described their everyday lives, their attention was directed toward activities that any child would do. They emphasised what they enjoyed and felt competent in rather than needs related to their health condition or healthcare follow-up. Technology was a largely taken-for-granted part of the children’s everyday lives, supporting gaming, schoolwork, social interaction and communication through mobile phones, tablets, gaming consoles and school-based digital tools. In contrast, they had limited experience using the same technologies as part of their healthcare services or follow-up, whether to communicate with healthcare professionals or to enable them to stay at home rather than being hospitalised.

Children had experience with home-based schooling, particularly after surgery, when their health condition deteriorated, or when prolonged home recovery was required. Home-based schooling was arranged through teacher home visits, participation in digital-based lessons or a combination of the two. The experiences with alternative teaching methods were generally described as positive, although they emphasised that these solutions could not fully replace being physically present in the classroom. As one child explained: ‘… It was fine, sometimes a bit impractical. It’s not always exactly the same things happening at school as at home. Like, they can do an assignment, but then I have to do it differently at home…’ (13-year-old child).

Another child used a school robot to attend classes digitally during infection risk periods in the COVID-19 pandemic, which was described as useful, even though it was accompanied by practical limitations. For example, in some cases, the child could see and hear the classroom but was not given the opportunity to speak. Certain tasks also had to be completed differently at home than at school; for example, some children mentioned home-based or school-based physiotherapy delivered by the municipal service. One child said she became tired after physiotherapy sessions scheduled immediately after school and would have preferred to rest, illustrating that a home-based consultation was not her preferred option. Another child considered exercising with a physiotherapist via screen but found it more motivating to train together in a gym setting. These accounts show that children’s physiotherapy experiences varied by setting and format, reflecting individual preferences for when and how sessions should be carried out.

### Technology has the potential to be a digital bridge to normalcy and social belonging

The children placed considerable importance on friends, family and school in their everyday lives. During prolonged periods at home or with reduced opportunities to be at school or with friends during home-based care, they used technology to maintain social contact and stay engaged with schoolwork and friends. Therefore, technology was described as an important tool for preserving social relations and a sense of belonging. Longer periods of absence from peers were described as boring and lonely, and they relied on phone calls, video calls and messaging apps to stay connected. Digital communication was an important way to participate in social life and maintain a sense of normality during these periods. The platforms used for this included various commercial social media, messaging and gaming applications.

All children attended regular schools, requiring varying degrees of adaptation, and school was a crucial arena for fostering belonging. The children were motivated to keep up academically and to remain connected with their classmates. Several had participated in digital lessons during periods of illness or absence, using diverse digital educational platforms. One participant suggested that a dedicated application for communication during periods of home-based care could be useful if it ensured secure login and protected privacy. This illustrated that children could envision digital solutions tailored to their needs when everyday routines were disrupted: ‘… Some kind of platform that parents and others can easily log into, a website or an app. And then people can have different usernames and stuff. It doesn’t take that long to make an email, it takes 2–3 min… Anyway, it would be easy to talk to each other like that. That it was an app or a website…’ (13-year-old child).

The children also emphasised their need to accommodate their individual preferences and fluctuating energy levels; some preferred video, others favoured audio and some chose text-based communication, depending on their condition. Ease of use and adaptability were highlighted as necessary for digital solutions to function well.

### Seeking information and safety through in-person meetings

The children indicated that their parents usually led conversations in consultations with healthcare professionals, as these situations needed safety, predictability and clear information. They preferred in-person meetings and relied on their parents to communicate with the healthcare professionals and to take care of the follow-up, although they felt capable of expressing themselves when needed. One child shared a school experience in which she struggled to communicate her wheelchair needs and felt dismissed by school staff and healthcare professionals, highlighting that children’s participation in decision-making was not always guaranteed. The children regularly attended hospital consultations and were familiar with the travel required for these appointments. Several described these trips not as a burden but sometimes as enjoyable because they offered a break from everyday routines and opportunities, such as going to a café, shopping or attending a football match. This contributed to in-person consultations being something they looked forward to, although with mixed feelings, as the consultations could include uncomfortable procedures or conversations. Their familiarity with travelling and time-consuming consultations underscored the necessity of their care and showed the children’s adaptability in managing their health follow-up.

Although this study, also reflected in the interview guide, aimed at exploring home-based care, many of the children had little experience with it or were unable to relate the follow-up they had received at home as home-based care. Their descriptions of meetings and communication with healthcare professionals were often related to hospital visits, and what concerned the children most were consultations involving injections, blood tests and surgeries. These experiences were associated with fear, pain and uncertainty. The children also expressed concerns about future surgeries, even when they recognised that such procedures might be necessary. They reported unclear communication about what procedures they would undergo and when. Clear information and preparation from healthcare professionals were described as important for managing these concerns. One child explained how his doctor accommodated his fear of needles and communicated that injections would be limited to those strictly necessary, which made the situation more manageable: ‘… But when I get a shot, I prefer to have that cream on [numbing cream]. And then I have to take off the band-aid… And then I don’t need to get it so often. Since I’m so afraid of needles, I didn’t have to get a shot, my doctor said. He said that when you’re this scared, then you don’t need to get a shot…’ (14-year-old child).

Together, the children’s descriptions illustrate that in-person meetings require supportive structures, parental involvement and clear, tailored communication from healthcare professionals. Their accounts of procedures, fears and uncertainty highlight that consistent and comprehensible information is essential for helping them feel safe and prepared.

## Discussion

We explored what children with LL/LT conditions find important in communication with healthcare professionals and others and the role of technology in daily life. The findings suggest that maintaining social relationships, participating in everyday activities and preserving a sense of normality were more important to the children than communicating with healthcare professionals through technology. The children also expressed a strong preference for in-person interactions with healthcare professionals, emphasising the importance of safety, predictability and clear information, particularly in situations involving fear or uncertainty.

A key finding in our study concerns the importance of social interaction and maintaining relationships between the child and friends, school and family during periods of illness, when they need to stay away for longer periods. These findings are consistent with Coombes *et al*^12^, who observed that while parents often focused on practical aspects of care, such as respite, logistics and service availability, children placed greater emphasis on staying at home, seeing friends and resuming everyday activities.^12^ In our study, the children demonstrated pragmatic and creative thinking around solutions to maintain these connections, such as proposing a secure communication application to prevent isolation during long periods at home or hospital stays. Studies have shown that technology-mediated social interaction can reduce emotional distress and improve well-being among hospitalised children.^28 29^

In our study, the children also emphasised that such tools must be adapted to developmental needs, privacy concerns and individual communication preferences (eg, video, voice or text). Previous studies have illustrated that parents and healthcare professionals share similar concerns regarding user-friendly, individually adapted health technology, as well as ensuring security and privacy when using such technology.^19 20 30^ We found that children primarily associate technology with schoolwork and social activities rather than home-based care. This may reflect limited exposure to and information about the possibilities for technology used in a healthcare context, as well as the dominance of adult-led decision-making in PPC, resulting in parents and professionals acting as gatekeepers.^18 26^ The children in our study described limited use of technology for direct communication with healthcare professionals. This suggests that there may be limited opportunities for children to engage directly with healthcare professionals through health technology. Despite increasing attention to the use and potential of health technology,^18^ most previous PPC research relies on information from parents and healthcare professionals, which may hinder the development of follow-up that meets children’s real needs.^5 23^

Another barrier could be that available health technologies are primarily designed for children with cancer and their families, with needs that often differ from other conditions eligible for PPC.^18^ In addition, much of the existing research on home-based care tends to concentrate on end-of-life care within the PPC.^10 31^ However, our focus was not end-of-life care but the everyday experience of living with an LL/LT condition, which reflects the circumstances of most children eligible for PPC.^4 32^ Research has suggested that children, including those as young as 8 years old, are capable of meaningful self-reporting when appropriate methods are used, underlining that children’s preferences regarding home-based care and the use of health technology cannot be assumed but must be elicited directly.^23^ Children’s limited awareness of options for home-based care and health technology, in combination with minimal involvement in care decisions, highlights the need for actions from healthcare professionals to actively engage children as informed contributors to their own care. Future studies should include children with LL/LT conditions in the development and implementation of health technology in home-based PPC.

Our findings align with family-centred theory principles, which emphasise that children and their families should be recognised as active partners in care.^7^ A family-centred approach highlights the importance of trust, shared decision-making and developmentally appropriate information to help children feel safe and involved. The need for predictability, limited participation in decision-making and adults’ gatekeeping underscore the importance of family-centred practices to ensure that children’s perspectives influence care planning.^7 18^

Despite the framing of the interviews being how technology can support communication with healthcare professionals for their healthcare follow-up, the children repeatedly voiced their concerns about medical procedures, described as painful. The children in our study described fear, pain and uncertainty regarding medical procedures, indicating a need for truthful age-appropriate information and preparation from healthcare professionals. These findings support the call for open, honest communication that positions the child as an active participant in their care.^22 29^ Previous research suggests that digital communication platforms and child-friendly health technologies, such as applications and video games, may serve as complementary tools to support preparatory conversations with children.^20 33^ Such technologies can help structure information, enhance predictability and offer customised ways for children to communicate with healthcare professionals and express concerns at their own pace.^20 33^ Technology in a health communication setting, and when designed with children’s perspectives in mind, may further strengthen these strategies by providing consistent and appropriate information and opportunities for children to articulate worries outside often time-pressured clinical consultations. Such strategies not only reduce distress but also foster a sense of self-determination and involvement that is often lacking for children living with complex, serious conditions.^23 29^

## Strengths and limitations

A key strength of this study lies in its focus on children’s own views, which are often underrepresented in PPC research.^5 23 26^ The children participating had limited experience with home-based care and the use of technology in a healthcare setting. This may be related to the inclusion of participants from only one health region, although children from all regions in Norway were invited to participate. Given the variations in service delivery and the integration of health technology across Norway,^34^ children in other regions may have broader or different experiences with digital home-based care. While including participants from additional regions might have increased variation, the existing dataset provided sufficient analytical depth and richness to illuminate children’s perspectives. In line with reflexive thematic analysis, the adequacy of the sample was evaluated based on the richness and relevance of the data in relation to the study aim rather than numerical considerations.^27^ Nevertheless, the children’s reflections remain valuable in highlighting their priorities and needs and provide important insights into how healthcare services can be better aligned with their everyday realities, regardless of regional differences.

Conducting only one interview per child may have limited the children’s opportunities to share openly, as some children may require time to feel safe and comfortable in an interview situation. Follow-up interviews might also have enabled additional reflections and facilitated greater openness, as prior familiarity with the interviewer may have increased the children’s comfort and willingness to share.^23^ The study demonstrates transparency by clearly describing the data collection process, analytical procedures and theme development. By documenting how data were generated, coded and interpreted through reflexive thematic analysis, the study enhances credibility and allows readers to assess the coherence between the data and the reported findings.^27^

## Conclusion

Children with LL/LT conditions primarily framed their experiences in relation to everyday participation, social relationships and maintaining a sense of normality. They emphasised the importance of feeling safe, well-informed and prepared for medical procedures, which contributed to their preference for in-person interactions with healthcare professionals. The children primarily associate technology with everyday social and school activities, rather than technology for home-based care or healthcare follow-up. Technology played a central role in sustaining normalcy and children’s social connections during periods of illness or absence from school, family and friends, and the children demonstrated pragmatic ideas for digital solutions that could prevent isolation. These findings highlight the importance of acknowledging children with LL/LT conditions’ technological competence and leveraging it to support communication, predictability and participation in their follow-up.

Overall, the findings underscore the need to involve children directly in the development and implementation of home-based PPC and communication technology solutions. Ensuring age-appropriate communication and integrating health technology in ways that align with children’s everyday technology practices may strengthen their sense of normalcy and social belonging. Therefore, future research should pay greater attention to children’s perspectives on communication, participation, and the role of health technology within their healthcare follow-up.

## Supplementary material

10.1136/bmjpo-2026-004998 online supplemental file 1

## Data Availability

All data relevant to the study are included in the article or uploaded as supplementary information.
