# Supplementary material for: Communication, everyday life and technology from the perspectives of children with life-limiting or life-threatening conditions: a qualitative study
Source: BMJ Paediatr Open. 2026 Sep 15;10(1):e004998. doi: 10.1136/bmjpo-2026-004998 (PMC13583832; doi:10.1136/bmjpo-2026-004998)
Supplement: Supplementary file 1 [file bmjpo-2026-004998.reviewer_comments.pdf]

PEER REVIEW HISTORY

BMJ Paediatrics Open publishes all reviews undertaken for accepted manuscripts. Reviewers are asked to complete a checklist review form and are provided with free text boxes to elaborate on their assessment. These free text comments are reproduced below.

ARTICLE DETAILS

**TITLE (PROVISIONAL)**	Communication, everyday life, and technology from the perspectives of children with life limiting or life threatening conditions—A qualitative study
**AUTHORS**	Martinsen, Linda<br>Holmen, Heidi<br>Steindal, Simen<br>Winger, Anette

VERSION 1 – REVIEW

**REVIEWER NAME**	LOBATON-SANABRIA, PAULA-ANDREA
**REVIEWER AFFILIATION**	
**REVIEWER CONFLICT OF INTEREST**	No Conflict of Interest
**AI DISCLOSURE**	No, I have not used AI
**DATE REVIEW RETURNED**	17-Jun-2026

**GENERAL COMMENTS**	The aim of this study is exploratory, and I have two questions regarding the analysis. I understand that the analysis and categories were developed inductively and emerged from the data. However, the way these categories are organized appears to follow a structured framework. How did the researchers determine when themes were sufficiently developed or saturated, and how was the relevance of each theme established? Additionally, could you clarify the process of triangulation among the researchers and how disagreements or differences in interpretation were resolved?

**REVIEWER NAME**	Pedraza, Eddy Carolina
**REVIEWER AFFILIATION**	Institut de Recerca Sant Joan de Deu
**REVIEWER CONFLICT OF INTEREST**	No Conflict of Interest
**AI DISCLOSURE**	So far, I have used Copilot primarily to improve grammar and writing style, and not for other purposes.
**DATE REVIEW RETURNED**	28-Jun-2026

**GENERAL COMMENTS**	Thank you very much for your manuscript. This is a highly relevant and timely topic, given the increasing use of technology in healthcare. Understanding the perspectives of children with life-limiting or life-threatening conditions is essential to provide comprehensive, needs-based care, both in hospital settings and at home.<br><br>I have a few suggestions:<br>1. The first concerns the title and the main objective. Although your stated aim was “to find what children consider important in

	communication with healthcare professionals and how they view the use of technology to support communication in their follow-up," the results appear to address communication in more general terms, rather than specifically with healthcare professionals. While this reflects your findings, it may be helpful to adopt a more general formulation for the title, as I expected more specific insights into communication with healthcare professionals, but in the current manuscript, this aspect seems less prominent, which may reflect either limited exploration or how the topic was understood by the participants (during the interview). Instead, the findings appear to place greater emphasis on communication with peers, within school settings, and at the community level (by using technology as a good tool for daily life activities).<br>In this regard, I would suggest something along the lines of: (this is only an example): "Perspectives of Children with Life-Limiting or Life-Threatening Conditions on the Role of Technology in Daily Home Care and the Importance of Communication with Healthcare Professionals and Others: A Qualitative Study"<br><br>2. Again, I particularly appreciate the results highlighting the high value placed on in-person communication with healthcare professionals, emphasizing the need for safety, predictability, and clear information, especially in the context of medical procedures. However, it would be worthwhile to more fully capture the range of other types of communication expressed by the children.<br>As currently presented, the findings may suggest that forms of technology-mediated communication with healthcare professionals are very limited, or even absent. This may not fully reflect the reality, although it is also possible. In this sense, it could represent a valuable result in itself, indicating that children may not be actively using technological tools to communicate directly with healthcare professionals, even if such tools are being used by parents or providers. This interpretation could open an important line of discussion regarding the need to better inform and empower children about available communication tools, and to promote more direct engagement between children and healthcare professionals through technology. At the same time, this may also explain why I experienced some confusion while reading the manuscript as a whole.<br><br>More specific suggestions:<br>The line numbering in the document is difficult to follow (throughout the manuscript - PDF); however, I will try to provide comments for each section:<br><br>Methods<br>Recruitment and Participants:<br>Page 9, lines 12–13: I do not fully understand what is meant by the following sentence:<br>"With consent from parents and children, healthcare professionals could also register interest on behalf of the children."<br>Does this imply that healthcare professionals could also participate?<br><br>Page 9, lines 20–21:<br>"If the parents found their child eligible, they discussed and clarified their participation with their child."<br>Does this mean that parents explained the study to the children? Was the study also explained to them by the researcher afterward?<br><br>Page 9, lines 30–31: What do you mean by "alternative or

	supplementary communication"? Could you provide an example/examples?<br><br>Data Collection<br>Page 10, line 19: "The guide was reviewed": what exactly do you mean by "reviewed"? Was the interview guide sent to one or more CHIP network representatives? How many were parents and how many were healthcare professionals?<br><br>Page 10, line 31: I would suggest rephrasing as: "referred to as follow-up at home," or "home follow-up," or "follow-up after discharge." (because it could also mean follow up at the hospital setting).<br><br>Page 11, lines 6–7: It states: "The pilot interview was conducted in the children's homes, with one of their parents present in the same room."<br>This should be singular:<br>"The pilot interview was conducted in the child's home, with one of his/her parents present in the same room."<br><br>Page 11, lines 11–12: "One of the children brought a friend" , was this friend an adult?, another child?<br><br>Page 11, lines 12–13: "Parents were not given specific guidance or instructions regarding what information the children received about the study in advance or how the interviews should be presented to them."<br>Is this correct? Do you think parents should have had more information about the study, the interview guide, and the types of questions that would be asked to their children? I consider they should have had.<br><br>Page 11, lines 40–41: "(see Table 1 for a description of the analysis)."<br>I would suggest writing:<br>Table 1 describes the six phases of the analysis based on Braun and Clarke's framework of reflective thematic analysis.<br><br>Preunderstanding<br>Page 13, lines 11–12: Please revise the sentence: "…for example, clinical communication and examination of the children and guidance and group services for guardians and siblings."<br>There is a duplication/triplication of the "and"; a comma before "group services" would improve clarity.<br><br>Page 13, lines 17–18: Instead of "The experience," it could be "Her experience," since it refers to LJM.<br><br>Ethical Considerations: You could add: Informed consent forms were signed.<br><br>Patient and Public Involvement: This section could be placed before the Ethical Considerations section.<br><br>Results<br>In general: Could the ages of the children be presented as frequencies? This would help to identify whether most participants were older or younger than 12 years (i.e., whether they were mainly adolescents or not). Age may have influenced the responses and,

	consequently, the results of this study. Maybe older children have known more about technology (in the healthcare context) than younger ones.<br><br>I understand that diagnoses may not be provided; however, could you include general categories of conditions (e.g., neurological, oncological, cardiovascular)? This could be presented at a broad level to avoid identification of participants.<br><br>Page 15, lines 15–16 and 21–22: Please revise for repetition: “Their use of technology seemed largely taken for granted, mainly via mobile phones, gaming consoles, tablets, and school-based digital tools. Using technology emerged as something the children took for granted, rarely considering it noteworthy.”<br><br>Page 16, lines 3–4: How many children? All of them?<br>“…children mentioned home- or school-based physiotherapy delivered by the municipal service.”<br><br>Page 16, lines 35–36: Please include “at school” in the sentence: (if you agree it suits)<br>“Longer periods of absence at school were …”<br><br>Discussion<br>Page 20, lines 43–46: I do not fully understand this sentence in the flow of the paragraph:<br>“However, we focused on the phase of living with a LL/LT condition, as the majority of children requiring PPC do (4, 32).”<br><br>Page 22, lines 13–14: I would suggest adding a subtitle such as “Strengths and Limitations”, and revising the paragraph to improve organization. Since you are mixing both.<br><br>Page 22, lines 18–20:<br>“…which may be related to the inclusion of participants from only one health region.”<br>Could you clarify why only one region was included in the results, if all regions were invited?<br><br>Page 22, lines 38–39: Please add “PPC”: “…how PPC services…” , or does it means all general services?<br><br>Conclusion<br>Page 23, lines 22–25: I suggest to replace “not” with “rather than”: “The children primarily associated technology with everyday social and school activities, rather than home-based …”<br><br>Page 23, lines 48–50: Is this referring specifically to follow-up at home or more generally?<br>“…with health technology within their follow-up.”<br><br>In the section competing interest also add non-conflic of interest.<br><br>Thank you again for your great work.

VERSION 1 – AUTHOR RESPONSE

Reviewer 1 comments	Authors’ responses

I understand that the analysis and categories were developed inductively and emerged from the data. However, the way these categories are organized appears to follow a structured framework. How did the researchers determine when themes were sufficiently developed or saturated, and how was the relevance of each theme established?<br><br>Additionally, could you clarify the process of triangulation among the researchers and how disagreements or differences in interpretation were resolved?	Thank you for this important comment.<br><br>The analysis was inductive. We have expanded the description of the analytic process to clarify how themes were developed and refined (in Table 2, phases 4 and 5). In line with Braun and Clarke's reflexive thematic analysis, we did not aim for data saturation. Rather than seeking data saturation, consistent with reflexive thematic analysis, we focused on developing themes that provided a rich and meaningful account of the children's perspectives and adequately addressed the research aim.<br><br>We have also clarified that interpretations and theme development were discussed within the research team throughout the analysis process. Differences in interpretation were explored through reflexive dialogue and critical discussion until a shared understanding of the data was reached. The revision can be found on pages 11-12 and has been highlighted in red:<br><br>*We actively sought to incorporate diverse perspectives and critically reflected on how our preconceptions shaped the research process. We did not seek consensus and differences in interpretation among us were explored through discussion and reflexive dialogue that enabled us to move beyond our preconceptions. Rather than seeking data saturation, consistent with reflexive thematic analysis, we focused on developing themes that provided a rich and meaningful account of the children's perspectives and adequately addressed the research aim.*

Reviewer 2 comments	Authors' responses
The first concerns the title and the main objective. Although your stated aim was "to find what children consider important in communication with healthcare professionals and how they view the use of technology to support communication in their follow-up," the results appear to address communication in more general terms, rather than specifically with healthcare professionals. While this reflects your findings, it may be helpful to adopt a more general formulation for the title, as I expected more specific insights into communication with healthcare professionals, but in the current manuscript, this aspect seems less prominent, which may reflect either limited exploration or how the topic was understood by the participants (during the interview). Instead, the findings appear to place greater emphasis on communication with peers, within school settings, and at the community level (by using technology as a good tool for daily life activities).<br>In this regard, I would suggest something along the lines of: (this is only an example):<br>"Perspectives of Children with Life-Limiting or Life-Threatening Conditions on the Role of Technology in Daily Home Care and the Importance of Communication with Healthcare Professionals and Others: A Qualitative Study"	Thank you for this thoughtful comment. We agree that the findings extend beyond communication with healthcare professionals and include broader aspects of social participation, everyday life, and technology use. To better align the title with the findings, we have revised the title and aim:<br><br>Title:<br>*Communication, everyday life, and technology from the perspectives of children with life-limiting or life-threatening conditions—A qualitative study*<br><br>Aim:<br>*This study aimed to explore what children with life-limiting or life-threatening conditions find important in communication with healthcare professionals and others, and the role of technology in daily life.*<br><br>We have also expanded the Discussion to explain how these broader narratives represent an important reflection of children's own priorities.<br><br>The revision can be found on page 18-22, and has been highlighted in red.
Again, I particularly appreciate the results highlighting the high value placed on in-person communication with healthcare professionals, emphasizing the need for safety, predictability, and clear information, especially in the context of medical procedures. However, it would be worthwhile to more fully capture the range of other types of communication expressed by the children.<br>As currently presented, the findings may suggest that forms of technology-mediated	Thank you for a very insightful comment. We agree that the children's limited descriptions of technology-mediated communication with healthcare professionals may constitute an important finding in itself. We have therefore expanded the discussion to acknowledge that the apparent absence of such communication may reflect limited awareness, opportunities, or involvement of children in technology-supported healthcare communication. We also

communication with healthcare professionals are very limited, or even absent. This may not fully reflect the reality, although it is also possible. In this sense, it could represent a valuable result in itself, indicating that children may not be actively using technological tools to communicate directly with healthcare professionals, even if such tools are being used by parents or providers. This interpretation could open an important line of discussion regarding the need to better inform and empower children about available communication tools, and to promote more direct engagement between children and healthcare professionals through technology. At the same time, this may also explain why I experienced some confusion while reading the manuscript as a whole.	clarify that this finding may help explain why children primarily discussed technology in relation to social participation, school, and everyday life rather than healthcare follow-up.<br><br>The revision can be found on page 18-22, and has been highlighted in red.
Recruitment and Participants:<br>Page 9, lines 12–13: I do not fully understand what is meant by the following sentence:<br>"With consent from parents and children, healthcare professionals could also register interest on behalf of the children."<br>Does this imply that healthcare professionals could also participate?	Thank you for pointing this out. We agree that the wording may be unclear. Healthcare professionals were not eligible participants in the study. Rather, with prior consent from both parents and children, healthcare professionals could inform the research team that a child was interested in participating.<br><br>We have revised the sentence to clarify this, and the revision can be found on page 8:<br>*With consent from parents and children, healthcare professionals could register interest in participation on behalf of the children.*
Page 9, lines 20–21:<br>"If the parents found their child eligible, they discussed and clarified their participation with their child."<br>Does this mean that parents explained the study to the children? Was the study also explained to them by the researcher afterward?	Thank you for this comment. We agree that the description was unclear. Parents initially discussed the study with their child and helped explain what participation would involve. Subsequently, the researcher provided age-appropriate information about the study and answered any questions before assent/consent was obtained. We have revised the text to clarify this procedure:<br><br>*At the start of every interview, the children received age-appropriate information from LJM about the study, its purpose, and an introduction to the interview topics .*

	Page 11.
Page 9, lines 30–31: What do you mean by "alternative or supplementary communication"? Could you provide an example/examples?	Thank you for this comment. We agree that the term may be unfamiliar to some readers. We have revised the text to provide examples of alternative or supplementary communication methods used, and the revision can be found on page 8:<br><br>*… and of being able to express themselves verbally or through alternative or supplementary communication (e.g., communication boards or pictorial symbols).*
Data Collection<br>Page 10, line 19: "The guide was reviewed": what exactly do you mean by "reviewed"? Was the interview guide sent to one or more CHIP network representatives? How many were parents and how many were healthcare professionals?	Thank you for this comment. We have clarified the description of the review process. The interview guide was distributed to members of the CHIP research network for feedback on the relevance, clarity, and comprehensibility of the questions. The aim of the manuscript is not to describe this composition; therefore, we have limited the description to the characteristics of the network rather than providing the exact number of parents and healthcare professionals who contributed comments. The text has been revised accordingly (page 10):<br><br>*The interview guide was distributed to all members of the CHIP research network, giving them the possibility for feedback on the relevance, clarity, and comprehensibility of the questions. .*
Page 10, line 31: I would suggest rephrasing as: "referred to as follow-up at home," or "home follow-up," or "follow-up after discharge." (because it could also mean follow up at the hospital setting).	Thank you for this helpful suggestion. We agree that terms such as "home follow-up" or "follow-up at home" would provide a more precise description and better distinguish the setting from hospital-based follow-up. However, when developing the interview guide, we deliberately used the broader and more familiar term "follow-up" because feedback from the pilot interview, HCP and user representatives indicated that simpler and less technical language was easier for children to understand and relate to. To remain faithful to

	the wording used in the interviews, we have therefore retained this term in the manuscript.
Page 11, lines 6–7: It states: “The pilot interview was conducted in the children’s homes, with one of their parents present in the same room.”<br>This should be singular:<br>“The pilot interview was conducted in the child’s home, with one of his/her parents present in the same room.”	Thank you for this suggestion, we have revised it to:<br><br>*The pilot interview was conducted in the child’s home, with one of his/her parents present in the same room.*<br><br>Page 10.
Page 11, lines 11–12: “One of the children brought a friend” , was this friend an adult?, another child?	Changed accordingly (page 10);<br><br>*One of the children brought a friend, who was also a child, while the other children came alone to the interviews.*
Page 11, lines 12–13: “Parents were not given specific guidance or instructions regarding what information the children received about the study in advance or how the interviews should be presented to them.”<br>Is this correct? Do you think parents should have had more information about the study, the interview guide, and the types of questions that would be asked to their children? I consider they should have had.	Thank you for this thoughtful comment. We acknowledge that providing parents with more detailed information or a standardized script for introducing the study to their children could have been beneficial. However, our rationale was to avoid influencing either parents’ or children’s perceptions and responses prior to the interviews and thereby allow children’s own experiences and perspectives to emerge as freely as possible. In retrospect, a more structured approach to the information provided to parents may also be considered a strength, as it could have ensured greater consistency in how the study was presented to potential participants.
Page 11, lines 40–41: “(see Table 1 for a description of the analysis).”<br>I would suggest writing:<br>Table 1 describes the six phases of the analysis based on Braun and Clarke’s framework of reflective thematic analysis.	Changed accordingly.<br><br>Page 11.
Preunderstanding<br>Page 13, lines 11–12: Please revise the sentence: “…for example, clinical communication and examination of the children and guidance	Changed accordingly.<br><br>Page 13.

and group services for guardians and siblings." There is a duplication/triplication of the "and"; a comma before "group services" would improve clarity.	
Page 13, lines 17–18: Instead of "The experience," it could be "Her experience," since it refers to LJM.	Changed accordingly.<br><br>Page 13.
Ethical Considerations: You could add: Informed consent forms were signed.	Thank you for this relevant suggestion. Information regarding informed consent procedures, including signed consent forms, is already described in the *Recruitment and Participants* section. To avoid repetition, we have chosen to retain this information in that section and not repeat it under *Ethical Considerations*.
Patient and Public Involvement: This section could be placed before the Ethical Considerations section.	Changed accordingly.<br><br>Page 13.
Results<br>In general: Could the ages of the children be presented as frequencies? This would help to identify whether most participants were older or younger than 12 years (i.e., whether they were mainly adolescents or not). Age may have influenced the responses and, consequently, the results of this study. Maybe older children have known more about technology (in the healthcare context) than younger ones.<br><br>I understand that diagnoses may not be provided; however, could you include general categories of conditions (e.g., neurological, oncological, cardiovascular)? This could be presented at a broad level to avoid identification of participants.	We appreciate this helpful suggestion. To provide additional context regarding the participant group while maintaining confidentiality, we have added information about the children's age distribution and broad life-limiting or life-threatening categories. Given the small sample size and the heterogeneity of diagnoses, we chose to describe participants according to internationally recognized life-limiting or life-threatening categories rather than diagnostic groups to reduce the risk of identification. In addition, the collection of participant characteristics was limited by the ethical approvals obtained from REK and Sikt, which also influenced the level of detail that could be reported.<br><br>Presented in Table 1, page 9.

Page 15, lines 15–16 and 21–22: Please revise for repetition:<br>"Their use of technology seemed largely taken for granted, mainly via mobile phones, gaming consoles, tablets, and school-based digital tools. Using technology emerged as something the children took for granted, rarely considering it noteworthy."	We agree that the wording was repetitive and the text has been revised to avoid repetition and changed accordingly (page 14).
Page 16, lines 3–4: How many children? All of them?<br>"…children mentioned home- or school-based physiotherapy delivered by the municipal service."	Thank you for this comment. We agree that the original wording was unclear. We have revised it to:<br>*Certain tasks also had to be completed differently at home than at school; for example, some children mentioned home- or school-based physiotherapy delivered by the municipal service.*<br><br>Page 15.
Page 16, lines 35–36: Please include "at school" in the sentence: (if you agree it suits)<br>"Longer periods of absence at school were …"	Thank you for this comment, we find it more suitable to use *… away from peers...*<br><br>Page 16.
Discussion<br>Page 20, lines 43–46: I do not fully understand this sentence in the flow of the paragraph:<br>"However, we focused on the phase of living with a LL/LT condition, as the majority of children requiring PPC do (4, 32)."	Thank you for this comment. We agree that the original wording was unclear. Our intention was to emphasize that, unlike much of the existing PPC literature, which often focuses on end-of-life care, our study explored children's experiences of living with an LL/LT condition during the broader illness trajectory. The sentence has been revised to clarify this point:<br><br>*However, our focus was not end-of-life care but the everyday experience of living with an LL/LT condition, which reflects the circumstances of most children eligible for PPC*<br><br>Page 20.
Page 22, lines 13–14: I would suggest adding a subtitle such as "Strengths and Limitations", and revising the paragraph to improve organization. Since you are mixing both.	Changed accordingly.<br><br>Page 22.
Page 22, lines 18–20:<br>"…which may be related to the inclusion of participants from only one health region."	Although recruitment was open nationwide, only children from one health region agreed to participate. We acknowledge that this may have limited the variation in experiences

Could you clarify why only one region was included in the results, if all regions were invited?	represented in the data and have clarified this limitation in the manuscript.
Page 22, lines 38–39: Please add “PPC”: “…how PPC services…” , or does it means all general services?	Thank you for this comment. We intended the term “services” to refer to healthcare services more broadly rather than specifically to PPC services. To avoid ambiguity, we have revised the wording in the manuscript to clarify this distinction:<br>*…and needs and provide important insights into how healthcare services can be better aligned with their everyday realities, regardless of regional differences.*<br><br>Page 22
Conclusion<br>Page 23, lines 22–25: I suggest to replace “not” with “rather than”:<br>“The children primarily associated technology with everyday social and school activities, rather than home-based …”	Thank you for this comment. We revised it to:<br>*The children primarily associated technology with everyday social and school activities, rather than technology for home-based care or healthcare follow-up.*<br><br>Page 23.
Page 23, lines 48–50: Is this referring specifically to follow-up at home or more generally?<br>“…with health technology within their follow-up.”	Thank you for this comment. We intended this statement to refer to healthcare follow-up more broadly rather than home-based follow-up specifically. To avoid ambiguity, we have revised the wording accordingly:<br><br>*Therefore, future research should pay greater attention to children’s perspectives on communication, participation, and the role of health technology within their healthcare follow-up.*<br><br>Page 24
In the section competing interest also add non-conflic of interest.	Changed accordingly. Page 24.

VERSION 2 – REVIEW

**REVIEWER NAME**	LOBATON-SANABRIA, PAULA-ANDREA
**REVIEWER AFFILIATION**	
**REVIEWER CONFLICT OF INTEREST**	No Conflict of Interest
**AI DISCLOSURE**	No, I have not used AI
**DATE REVIEW RETURNED**	08-Aug-2026

**GENERAL COMMENTS**	Ok. I agree

**REVIEWER NAME**	Pedraza, Eddy Carolina
**REVIEWER AFFILIATION**	Institut de Recerca Sant Joan de Deu
**REVIEWER CONFLICT OF INTEREST**	No Conflict of Interest
**AI DISCLOSURE**	Artificial intelligence tools were used solely to improve the clarity, grammar, and style of the review text. All intellectual contributions, including the scientific assessment, interpretations, critiques, and recommendations, were developed independently by the reviewer.
**DATE REVIEW RETURNED**	04-Aug-2026

**GENERAL COMMENTS**	Reviewer Comments (Minor Revisions)<br><br>Thank you very much for your detailed responses to my comments. I consider the manuscript has been substantially strengthened and enriched through the revisions made. The authors should be congratulated for their thoughtful work in addressing the previous concerns.<br>Before final acceptance, I have a few minor points for consideration:<br><br>Abstract<br>• In the third line, please add the word "and" between "coordinated" and "multidisciplinary":<br>"…which necessitates coordinated and multidisciplinary support…"<br><br><br>What This Study Adds and Conclusion<br>• At the beginning of the sentence, the term "children" may be interpreted as referring to all children in general. However, the findings relate specifically to the sample of children with LL/LT conditions included in this study. I would recommend clarifying this wording to avoid overgeneralization.<br><br><br>Recruitment and Participants<br>• In the fourth paragraph, please add "years old" after the age ranges:<br>"For children under 12 years old who agreed to participate, the parents provided digital consent on their behalf. For children between 12 and 16 years old…"<br><br><br>Ethical Considerations<br>• Please consider adding the following statement:<br>"The study was conducted in accordance with the Declaration of Helsinki and relevant national regulations."<br><br><br>Results<br>• Please consider whether Figure 1 is essential to the manuscript. The authors may wish to evaluate whether it adds substantial value beyond the text.<br><br>Understanding Technology Through Daily Life Rather Than in the Frame of Healthcare<br>• The following two sentences appear somewhat repetitive and could potentially be merged:

	"Technology was an integrated part of their daily routines through gaming, schoolwork, digital social meeting platforms, and communication with friends."<br>"Technology was an integrated and largely taken-for-granted part of the children's everyday lives, primarily through mobile phones, gaming consoles, tablets, and school-based digital tools."<br><br>Discussion<br>• The following statements also seem somewhat repetitive:<br>"The findings showed that the children's engagement with technology was primarily directed toward social and educational activities rather than communication with healthcare professionals."<br>"Their accounts suggest that everyday participation, social relationships, and maintaining a sense of normality were more salient in their lives than communication with healthcare professionals or healthcare-related technology."<br><br>Strengths and Limitations<br>• After line 4, I would suggest adding the following sentence:<br>"The children participating had limited experience with home-based care and the use of technology in a healthcare setting, which may be related to the fact that all participants came from a single health region, although children from all regions in Norway were invited to participate."<br><br>Conclusion<br>• As noted above, when referring to "children" at the beginning of the conclusion, the wording may be interpreted as applying to all children rather than the specific sample of children with LL/LT conditions included in this study. Consider revising the phrasing to avoid overgeneralization.<br>• The following two sentences could potentially be merged, as they convey closely related ideas:<br>"The children primarily associate technology with everyday social and school activities, rather than technology for home-based care or healthcare follow-up."<br>"Technology played a central role in sustaining normalcy and children's social connections during periods of illness or absence from school, family, and friends, and the children demonstrated pragmatic ideas for digital solutions that could prevent isolation."<br><br>Ethical Approval and Participant Consent<br>• Please also consider adding:<br>"The study was conducted in accordance with the Declaration of Helsinki and relevant national regulations."<br><br>Once again, I would like to congratulate the authors on the considerable improvements made to the manuscript. Subject to consideration of these minor points, I believe the manuscript is suitable for publication.

VERSION 2 – AUTHOR RESPONSE

Reviewer 2 comments	Authors' responses
**Abstract**<br>• In the third line, please add the word "and" between "coordinated" and "multidisciplinary":<br>"…which necessitates coordinated and multidisciplinary support…	Thank you. The word "and" has been added as suggested.<br><br>The sentence now reads (Abstract, background, second sentence: *"However, they prefer to live as normally as possible, which necessitates coordinated and multidisciplinary support that allows them to be at home rather than in a hospital."*
**What This Study Adds and Conclusion**<br>• At the beginning of the sentence, the term "children" may be interpreted as referring to all children in general. However, the findings relate specifically to the sample of children with LL/LT conditions included in this study. I would recommend clarifying this wording to avoid overgeneralization.	Thank you for this valuable comment. The wording has been revised to clarify that the findings relate specifically to children with LL/LT conditions included in this study.<br><br>The sentence now reads (What this study adds, first sentence): *"Children with life-limiting or life-threatening conditions associated technology mainly with everyday social interaction rather than healthcare and reported limited involvement in decisions about their care "*.
**Recruitment and Participants**<br>• In the fourth paragraph, please add "years old" after the age ranges:<br>"For children under 12 years old who agreed to participate, the parents provided digital consent on their behalf. For children between 12 and 16 years old…"	Thank you. The word "years old" has been added as suggested. The text now reads:<br><br>*"For children under 12 years old who agreed to participate, the parents provided digital consent on their behalf. For children between 12 and 16 years old, both the child and the parents signed a consent form"*
**Ethical Considerations**<br>• Please consider adding the following statement:<br>"The study was conducted in accordance with the Declaration of Helsinki and relevant national regulations."	We have added this statement to the Ethics section of the manuscript.
**Results**<br>• Please consider whether Figure 1 is essential to the manuscript. The authors may wish to	Thank you for this suggestion. After careful consideration, Figure 1 has been removed from the manuscript, as we agree that it adds limited

evaluate whether it adds substantial value beyond the text.	value beyond the information already presented in the text.
Understanding Technology Through Daily Life Rather Than in the Frame of Healthcare<br>• The following two sentences appear somewhat repetitive and could potentially be merged:<br>“Technology was an integrated part of their daily routines through gaming, schoolwork, digital social meeting platforms, and communication with friends.”<br>“Technology was an integrated and largely taken-for-granted part of the children’s everyday lives, primarily through mobile phones, gaming consoles, tablets, and school-based digital tools.”	Thank you for this observation. To reduce repetition and improve readability, the two sentences have been merged into a single sentence:<br><br>*“Technology was a largely taken-for-granted part of the children’s everyday lives, supporting gaming, schoolwork, social interaction, and communication through mobile phones, tablets, gaming consoles, and school-based digital tools”*
**Discussion**<br>• The following statements also seem somewhat repetitive:<br>“The findings showed that the children’s engagement with technology was primarily directed toward social and educational activities rather than communication with healthcare professionals.”<br>“Their accounts suggest that everyday participation, social relationships, and maintaining a sense of normality were more salient in their lives than communication with healthcare professionals or healthcare-related technology.”	Thank you for this observation. To reduce repetition and improve readability, the statements have been merged and revised:<br><br>*“The findings suggest that maintaining social relationships, participating in everyday activities, and preserving a sense of normality were more important to the children than communicating with healthcare professionals through technology.”*
**Strengths and Limitations**<br>• After line 4, I would suggest adding the following sentence:<br>“The children participating had limited experience with home-based care and the use of technology in a healthcare setting, which may be related to the fact that all participants came from a single health region, although children from all regions in Norway were invited to participate.”	Excellent suggestion! We agree that this is an important limitation to acknowledge and have added a revised version of the suggested text to the Strengths and Limitations section.<br><br>*“The children participating had limited experience with home-based care and the use of technology in a healthcare setting. This may be related to the inclusion of participants from only one health region, although children from*

	all regions in Norway were invited to participate"
**Conclusion**<br>• As noted above, when referring to "children" at the beginning of the conclusion, the wording may be interpreted as applying to all children rather than the specific sample of children with LL/LT conditions included in this study. Consider revising the phrasing to avoid overgeneralization.<br>• The following two sentences could potentially be merged, as they convey closely related ideas:<br>"The children primarily associate technology with everyday social and school activities, rather than technology for home-based care or healthcare follow-up."<br>"Technology played a central role in sustaining normalcy and children's social connections during periods of illness or absence from school, family, and friends, and the children demonstrated pragmatic ideas for digital solutions that could prevent isolation."	Thank you for this observation. We have added this to the conclusion section:<br>*"These findings highlight the importance of acknowledging children with LL/LT condition's technological competence and leveraging it to support communication, predictability, and participation in their follow-up".*<br><br>Thank you for this suggestion. While we appreciate the potential for reducing repetition, we have retained the two sentences as they emphasize different dimensions of the findings, and we believe this improves the clarity of the results.
**Ethical Approval and Participant Consent**<br>• Please also consider adding:<br>"The study was conducted in accordance with the Declaration of Helsinki and relevant national regulations."	Thank you for this suggestion. The statement has been added to the "Ethical Approval and Participant Consent" section.
