# Supplementary material for: Communication, everyday life and technology from the perspectives of children with life-limiting or life-threatening conditions: a qualitative study
Source: BMJ Paediatr Open. 2026 Sep 15;10(1):e004998. doi: 10.1136/bmjpo-2026-004998 (PMC13583832; doi:10.1136/bmjpo-2026-004998)
Supplement: Supplementary file 2 [file bmjpo-10-1-s001.docx]

**Interview guide – Introduction**

I will start by asking you a few questions about yourself and your everyday life. There are no right or wrong answers. You can tell me as much or as little as you like, and if there is something you do not want to answer, that is completely fine.

- Can you tell me about something you brought with you today? (The child brings an object, toy, or item that says something about who they are or tells a story about them).
- Can you tell me what a typical day is like for you?
- What do you usually do from the time you wake up until you go to bed?
- School, hobbies/leisure activities, friends.

**Interview guide – Main part**

Now I would like to ask you some questions about your illness/condition and about the support you receive in everyday life, for example at home or at school.

- First, can you tell me what you know about your illness/condition? (Using the child's own words)
- What kinds of things do you need to do because of your illness/condition?
- What kinds of help, treatment, training, or support do you receive?
- What do you find most helpful?
- What kind of support is most important to you?
- Have you received any support or follow-up that you did not like or did not find helpful?
- What made it unhelpful?
- Is there anything you currently go to the hospital for that you would rather receive or do at home?

Now I would like us to talk about these pictures (shows pictures).

Some people call this health technology or digital technology. What would you call it?

- What comes to mind when you look at these pictures?
- Do you use digital technology at home or at school?
- How do you use it?
- What do you like or dislike about using it?
- Do you use any digital tools that help people keep track of how you are doing or how your illness/condition is affecting you?

**If yes:**

- Can you tell me about it?
- What has that experience been like for you?

**If no:**

- Can you think of ways that digital tools might make it easier for you to stay at home instead of travelling to the hospital?
- What do you think about using digital tools to talk with people who work at the hospital?
- What do you think about sending or receiving messages, or having video calls with healthcare professionals?

I would also like to talk a little about the people who are important to you.

- Who are the people you like to talk to or spend time with?
- At home (family)?
- With friends or other relatives?
- At school (teachers, assistants, school nurse)?
- At the hospital?
- Anywhere else?
- If you could invent something that would help you stay in touch with these people when you are at home or in hospital, what would it be like?
- Would it include video calls, chat, games, questions, or something else?

**Interview guide – Closing**

We are now coming to the end of the interview.

- Is there anything important that we have not talked about that you would like to tell me?
- Is there a question you think I should have asked?

Summary of the interview/conversation.
